# Connecting the Kinetics and Energy Landscape of tRNA Translocation on the Ribosome

**DOI:** 10.1371/journal.pcbi.1003003

**Published:** 2013-03-21

**Authors:** Paul C. Whitford, Scott C. Blanchard, Jamie H. D. Cate, Karissa Y. Sanbonmatsu

**Affiliations:** 1 Department of Physics, Northeastern University, Boston, Massachusetts, United States of America; 2 Center for Theoretical Biological Physics, Rice University, Houston, Texas, United States of America; 3 Theoretical Biology and Biophysics, Theoretical Division, Los Alamos National Laboratory, MS K710, Los Alamos, New Mexico, United States of America; 4 Department of Physiology and Biophysics, Weill Cornell Medical College of Cornell University, New York, New York, United States of America; 5 Departments of Molecular and Cell Biology and Chemistry, University of California at Berkeley, Berkeley, California, United States of America; 6 Physical Biosciences Division, Lawrence Berkeley National Laboratory, Berkeley, California, United States of America; University of Missouri, United States of America

## Abstract

Functional rearrangements in biomolecular assemblies result from diffusion across an underlying energy landscape. While bulk kinetic measurements rely on discrete state-like approximations to the energy landscape, single-molecule methods can project the free energy onto specific coordinates. With measures of the diffusion, one may establish a quantitative bridge between state-like kinetic measurements and the continuous energy landscape. We used an all-atom molecular dynamics simulation of the 70S ribosome (2.1 million atoms; 1.3 microseconds) to provide this bridge for specific conformational events associated with the process of tRNA translocation. Starting from a pre-translocation configuration, we identified sets of residues that collectively undergo rotary rearrangements implicated in ribosome function. Estimates of the diffusion coefficients along these collective coordinates for translocation were then used to interconvert between experimental rates and measures of the energy landscape. This analysis, in conjunction with previously reported experimental rates of translocation, provides an upper-bound estimate of the free-energy barriers associated with translocation. While this analysis was performed for a particular kinetic scheme of translocation, the quantitative framework is general and may be applied to energetic and kinetic descriptions that include any number of intermediates and transition states.

## Author Summary

With a comprehensive understanding of the underlying energetics for a biomolecular machine, such as the ribosome, we may quantitatively identify the essential factors that determine function. In molecular-level machines, the energetic fluctuations arising from the surrounding solvent become comparable to the energetics that direct each process. In this regime, structural rearrangements may be described as diffusive movements across a free-energy landscape. By adopting this framework, it is possible to quantitatively relate theoretical predictions, computational results, experimental rates and biochemical data, which will allow for a self-consistent description to be obtained. To this end, we have used an explicit-solvent simulation (2.1 million atoms, 1.3 microseconds) to measure the diffusion along multiple structural coordinates that have been implicated in tRNA translocation in the ribosome. With the diffusion coefficients in hand, we demonstrate how they may be used to bridge theoretical and experimental descriptions of complex collective dynamics in a molecular machine.

## Introduction

**Figure 1:**
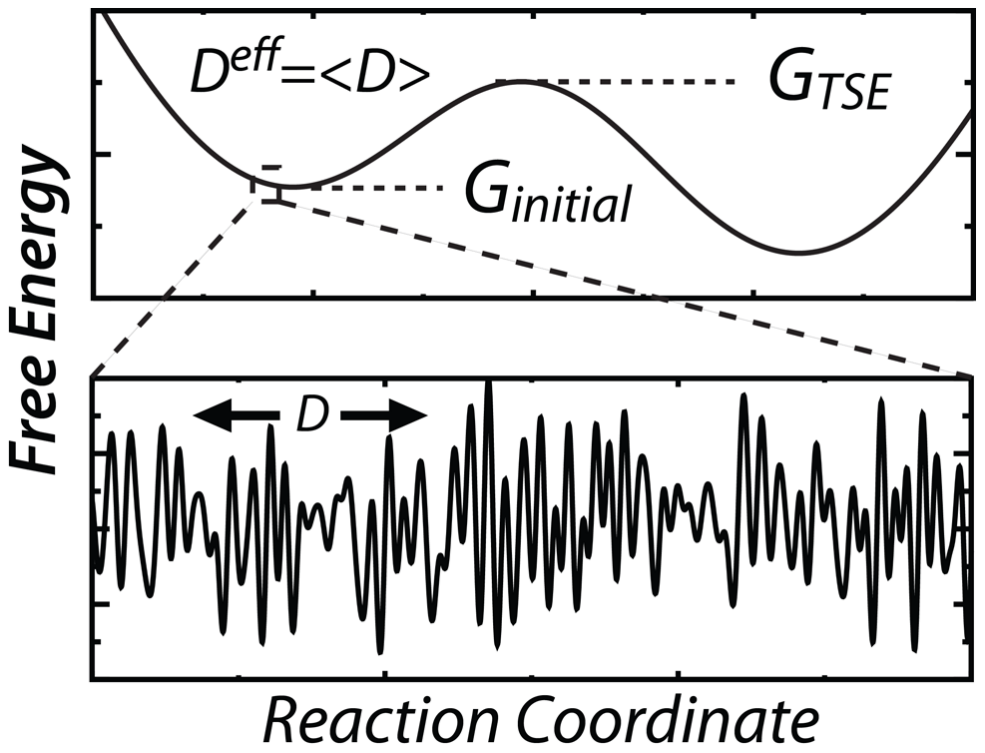
Energy landscapes. Short length-scale diffusion on a rough landscape 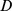 (bottom) may be averaged, yielding an effective diffusion 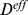, which is obtained from simulation. Effective diffusion leads to barrier crossing attempts, where the probability of crossing is governed by the height of the long length-scale barrier (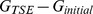).

Biological machines are ubiquitous in the cell and typically contain many molecules that include protein, RNA, and other cofactors. Each molecule provides a unique contribution to an assembly's energy landscape, which then governs the machine's function. Accordingly, quantifying the landscape's features and molecular origins may allow one to precisely manipulate the physical-chemical properties that dictate the biological dynamics. Despite the pressing need for a quantitative description of the energy landscapes that underpin function, most experimental techniques report on the rates of interconversion between states, where each state is a discretized approximation to an energetic basin. To bridge discrete and continuous descriptions for a molecular machine, such as the ribosome, it is necessary to quantify the diffusive properties of functionally-relevant collective rearrangements. The observed, or effective, diffusion of each component of a biomolecular complex is determined by the intrinsic diffusion of that component (free in solution) as well as the short-scale energetic roughness that is introduced by molecular interfaces [1]–[5]. In other words, the landscape is characterized by energetic barriers that are associated with a hierarchy of length scales (Fig. 1) [6], [7], where the effective diffusion is dictated by the magnitude of the short length-scale roughness. Structural rearrangements may then be described by effective (short length-scale averaged) diffusion on a smooth, large-scale, energy landscape. By measuring the effective diffusion, one may determine the relationship between the long length-scale free-energy barriers and the kinetics associated with interconversion between well-defined states.

**Figure 2:**
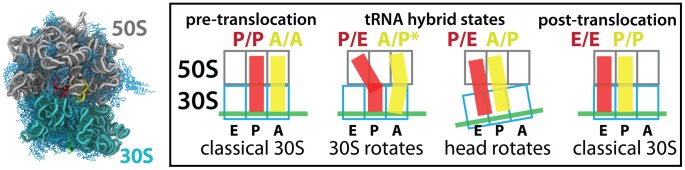
tRNA translocation. Explicit-solvent simulation of an E. coli ribosome (water molecules and ions not shown), colored by region: 23S/5S rRNA (gray), 16S rRNA (cyan), proteins (light blue), P/P tRNA (red) and A/A tRNA (yellow). During translocation, tRNA molecules adopt hybrid configurations (middle). Rotation of the 30S body (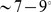) and head (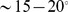) is associated with tRNA movement between binding sites. Here, we initiated the simulation in the pre-translocation configuration and characterized the structural fluctuations about the classical tRNA configuration and unrotated subunit configuration.

**Figure 3:**
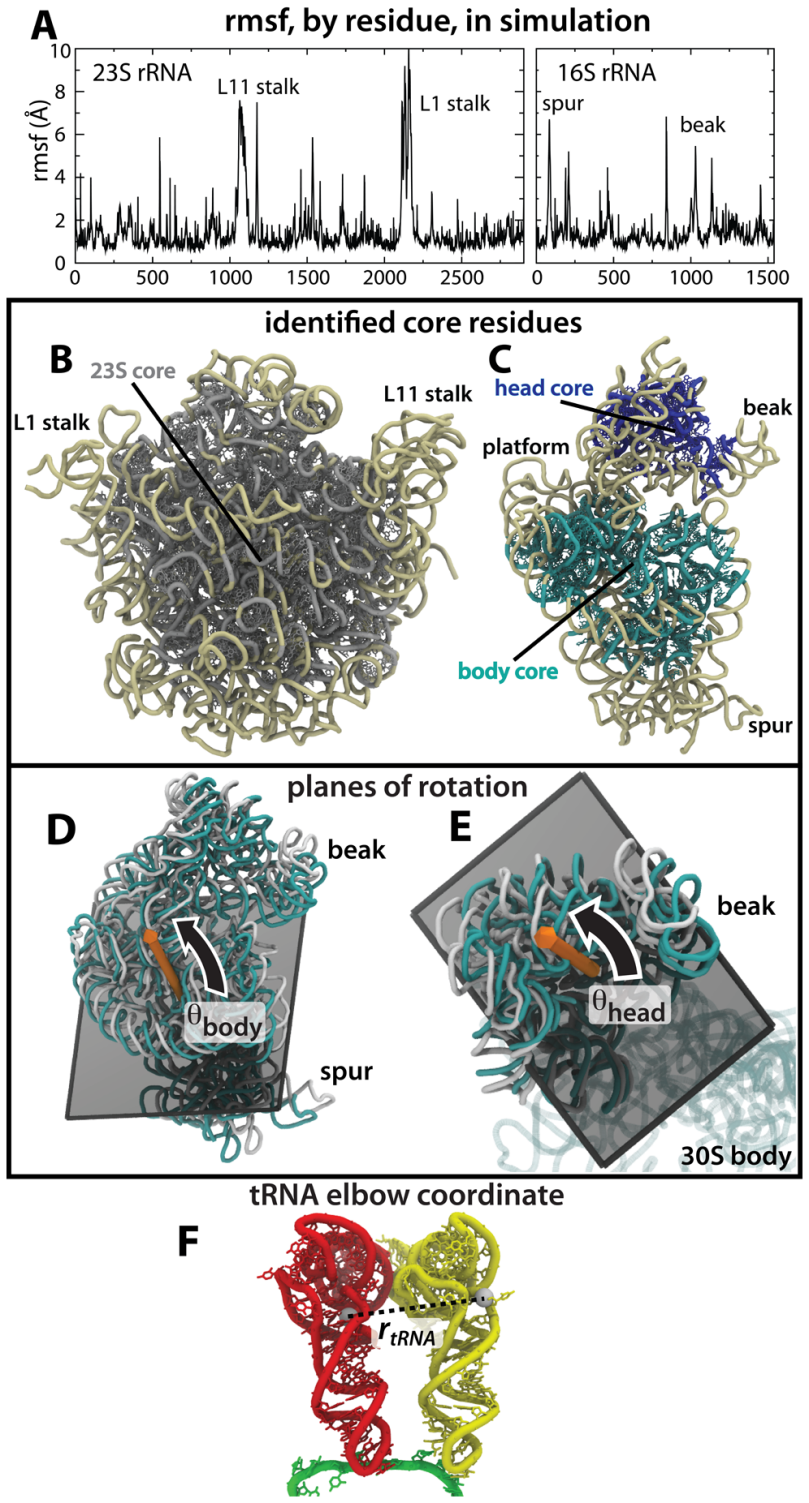
Reaction coordinates for 30S rotation and tRNA movement. A) rmsf, by residue, for the 23S (left) and 16S (right) rRNA. rmsf measures (See Text S1) were used to define the core residues (shown with side chains) of the: B) 23S rRNA (gray) and C) 16S body (cyan) and 16S head (blue). Core residue groups were used to define the planes of rotation for D) body rotation (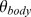, positive in the counter-clockwise direction) and E) head swivel (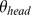). The vectors that define the rotation planes are depicted by orange arrows. The angle between the vectors is 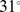. In (D) and (E), the classical and rotated configurations are shown in cyan and white. tRNA position is measured by 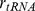, as defined previously [49], [50] and is shown for F) the classical A/A-P/P tRNA configuration.

**Figure 4:**
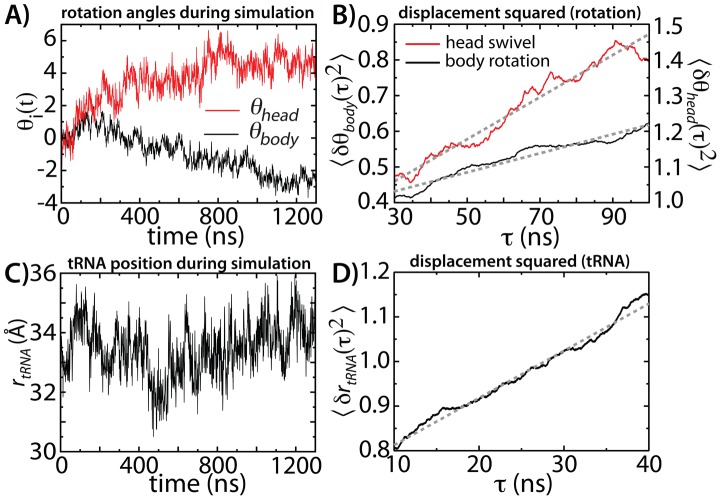
Estimating diffusion coefficients for subunit rotation and tRNA movement. A) Rotation coordinates 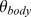 and 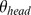 as functions of time for a simulation about the pre-translocation (A/A-P/P) configuration (every 1 ns shown). B) Effective diffusion coefficients 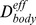 and 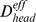 were obtained from the displacement squared in the angles 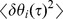. 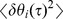 is linear for 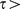
30 ns, which is characteristic of diffusive dynamics. Linear fits are depicted with gray dashed lines. C) 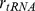 over the course of the simulation. D) Displacement squared of 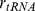 is linear for 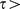
10 ns, allowing the tRNA effective diffusion coefficient 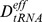 to be measured.

The ribosome has long been considered to function as a “thermal ratchet machine” [8], in that random energetic fluctuations that result from finite temperatures lead to large-scale diffusive (i.e. Brownian) configurational rearrangements. While smFRET and simulations directly monitor diffusive movement across the landscape, kinetic and structural measures utilize discrete state-like approximations to describe a molecule's dynamics. These seemingly disparate perspectives can be rationalized by adopting energy landscape theory [9], [10], which was developed in the context of protein folding and then extended to describe functional dynamics [11]–[15]. To this end, we have combined all-atom molecular dynamics simulations and principles from energy landscape theory to provide the quantities necessary to describe large-scale collective dynamics in the ribosome. To achieve this, we analyzed the dynamics of an explicit-solvent simulation of an intact ribosome to identify groups of residues that undergo collective rotations/displacements. From this, we identified collective reaction coordinates that capture 30S-body rotation (i.e. “ratchet-like” motion), 30S-head swivel and tRNA displacements (Fig. 2 and 3), which are essential motions during substrate translocation (i.e. the directional movement of tRNA and mRNA molecules, with respect to the ribosomal subunits; described in detail in Text S1) [16], [17]. In a continuous 1.3 microsecond explicit-solvent simulation of the pre-translocation complex, frequent small-scale body-rotation, head-swiveling and tRNA fluctuations were observed, from which effective diffusion coefficients were calculated in each coordinate space (Fig. 4). Full body and head rotation during translocation encompass angular displacements of 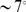 and 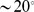 [18]–[23]. Smaller net rotations (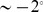 and 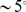) were observed in the simulation. Dynamics of the pre-translocation complex provided quantitative measures of the short time-scale (10–100 ns) fluctuations and effective diffusion in each space, which were used to relate the kinetics and free-energy barriers of translocation. From this analysis, we provide experimentally-grounded upper bounds for the long length-scale barriers associated with translocation, as well as estimates for the magnitude of the short-scale energetic roughness.

## Results

### Identifying collective coordinates for subunit rotation

To quantify the energy landscape of translocation, it is necessary to identify coordinates that are able to accurately capture these motions (which encompass large-scale rotary movement of the subunits). Structural approaches, such as x-ray spectroscopy and cryo-electron microscopy (cryo-EM), provide snapshots of the ribosome that describe the average configurations of energetic minima. These structural models provide tremendous insights into the global architecture of biomolecular assemblies, though the models can not separate fluctuations that are due to movement of individual residues from many-residue collective rearrangements. For example, x-ray and cryo-EM models have shown that the 30S subunit rotates relative to the 50S subunit [18]–[23]. From structural models of the endpoints, one may be inclined to think that movement is rigid-body like, since rigid atomic models may fit well to the endpoint configurations. However, biomolecular systems are constantly subject to energetic fluctuations that arise from the surrounding solvent [24], [25], leading to heterogeneous distributions of atomic fluctuations. Accordingly, some regions exhibit fluctuations in the coordinates that are faster than (and not coupled to) the global rearrangements [26]. Here, we use all-atom explicit-solvent simulations to ask: For tRNA translocation in the ribosome, can we decompose the process into a superposition of slow, large-scale collective movements and rapid local fluctuations? If so, what regions of the ribosome undergo each class of motions? As described below, we find that many residues undergo coordinated displacements, which are reminiscent of rigid body movement. In addition to the coordinated movement of these “rigid” groups, roughly half of the rRNA residues undergo independent fluctuations. These peripheral fluctuations are likely linked to functional capabilities and control of the ribosome, which may include the movement of ribosomal “stalks” during elongation [27]–[33], regulation of tRNA association and movement [34]–[38], antibiotic function [39] and ribosomal stalling [40].

To probe the energy landscape of 30S body and head rotation and tRNA displacements, we first identified reaction coordinates upon which to project the free energy. For an appropriate coordinate 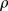 (here, 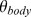, 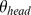, and 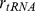), the potential of mean force (pmf) captures the scale and position of the multidimensional free-energy barrier. Then, 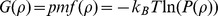, where 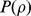 and 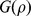 are the probability distribution and free energy, as functions of 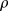. In order to describe body and head rotation as effective diffusion along an energy surface, each reaction coordinate  was required to satisfy the following minimal set of conditions: 1) for each biomolecular configuration, 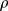 is uniquely defined; 2) 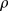 is a continuous function of the molecular coordinates; 3) collective rearrangements are measured by 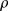 and independent fluctuations of individual atoms are not; 4) movement between the endpoints of translocation leads to changes in 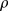, while orthogonal displacements do not; 5) the endpoints and the transition state ensembles (TSEs) correspond to distinguishable values of 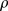; and 6) the dynamics in 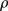-space is diffusive. In addition to these conditions, it would be desirable to also demonstrate that the dynamics along each coordinate is Markovian, and that the theoretical free-energy profile and diffusion coefficient (possibly coordinate-dependent) yield a rate that is consistent with the same simulated trajectory. While that form of analysis is computationally tractable for the folding of small proteins [41]–[46], it is not yet feasible for systems as large as the ribosome. However, as computational capacity [47] and methods [48] continue to develop, it may soon be possible to perform comparable analysis for large system. Nonetheless, at this point, we use the remaining conditions to provide evidence of the suitability for a specific set of coordinates for translocation, with which the relationship between barriers and kinetics are calculated. Since 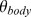 and 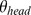 are continuous functions of the atomic coordinates, conditions 1 and 2 are met. Conditions 3–5 were ensured through analysis of an explicit-solvent simulation, crystallographic models and atomic models of cryo-EM reconstructions (Fig. 3; see Methods and Text S1). As discussed below, in the simulation, movement along 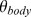 and 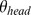 is diffusive in character, indicating that point 6 is also satisfied. 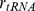 (called 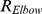 elsewhere) was previously shown to satisfy these considerations [49]. Together, these calculations provide systematically-identified coordinates for describing the diffusive body-rotation, head-swivel and tRNA displacements that occur during translocation.

To construct coordinates for 30S body and head rotation ( 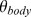 and 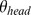) that measure collective rotations and not the fluctuations of individual atoms, we first identified groups of residues within each subunit that undergo minimal (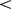
1 Å) internal structural fluctuations. This was accomplished through an iterative-exclusion strategy (See Methods) that is based on the spatial root-mean-squared fluctuations (rmsf) of each residue (Fig. 3A). Consistent with previous calculations [27]–[31], [50] and experimental measurements [51], large portions of the ribosome were relatively immobile in the simulation (i.e. small internal rearrangements), whereas peripheral regions underwent rapid, larger-scale structural fluctuations. Of the 2903 residues in the 23S rRNA of the 50S subunit, we identified 1353 residues as scaffolding, or “core,” residues. Similarly, in the 16S rRNA of the 30S subunit, of the 1060 30S-body residues considered, 443 were identified as being part of the core. Of the 284 30S-head residues considered, 178 were identified as core residues (Figs. 3 and S1; see Text S1 for list of residues). Consistent with our identification of the core residues, many have small anisotropic crystallographic B-factors [52], which measure the mobility of each atom [53]. A noticeable exception is the elevated B-factors of the 30S-head residues (Fig. S2). This may be attributed to the fact that the B-factors measure the total dispersion in the coordinates, where relative displacements of domains can elevate the B-factors, even if each domain is internally rigid. In the case of the 30S-head region, uncertainties in the relative orientation of the head, relative to the 30S body and the 50S are likely to elevate the B-factors. Through simulation, we avoid this effect by calculating the relative mobility of subsets of residues, which specifically isolates the internal fluctuations of each region.

Analysis of the fluctuations of each subregion (50S, 30S-body and 30S-head) indicates that large sets of atoms within each subunit maintain their structural integrity while the core group undergoes displacements relative to other subunits. Accordingly, the configurations of the cores residues were analyzed for a variety of experimental structural models, in order to identify the vectors of rotation that define 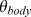 and 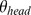 (Fig. 3; See Methods). Rather than measure the relative orientation of a single pairs of atoms in each model, which would be susceptible to the local fluctuations of each atom, collective rearrangement of each subunit was measured by first finding the average orientation of the core residues in each model. That is, even if every atom fluctuates in Cartesian space with the same length scale, the projection onto the 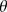 coordinates would depend on the distance between the atom pair used. Individual atomic fluctuations would then have differential effects on the rotation fluctuations, if the averaging step were not employed. To obtain the average, a reference model was fit to the core residues of each structural model. The rotation vector was then defined by finding the atom pair (within the rigid, fitted models) that has a maximal difference in angle between functional configurations (See Methods and Text S1 for details). With the rotation vectors identified, the rotation angles may be calculated for any experimental or computationally-generated configuration of the ribosome. Here, we provide the values of 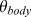 and 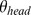 for a variety of available x-ray and cryo-EM models (Table S1), as well as for each frame of our simulated trajectory (10 ps intervals).

### Measuring diffusive dynamics

With the rotation angles calculated for each simulated frame, we next asked if the dynamics in these coordinate spaces is diffusive, or not. We found that the rotation coordinates and tRNA coordinate exhibit diffusive behavior (Fig. 4B/D), supporting their use as reaction coordinates for translocation. The displacement-squared along each coordinate scales linearly with time, at long time, such that effective diffusion coefficients along each can be obtained from the relation:
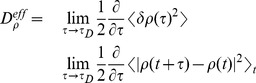
(1)where 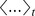 indicates an average over all simulated frames that are separated by a given value of 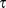, and 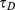 is the timescale associated with diffusive movement (i.e. where 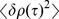 becomes linear). After an initial burst phase (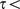
30 ns, Fig. S3), 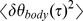 and 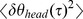 exhibit linear behavior, with linear correlation coefficients (c.c.) of 0.97 and 0.98. Similarly, after an initial burst phase of 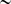
10 ns, 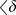 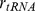 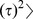 follows a linear trend (Fig. 4D), where c.c. = 0.99. This linear behavior is characteristic of diffusive dynamics, where the slopes of each fit indicate 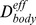 = 1.39 degrees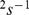, 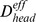 = 2.95 degrees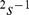 and 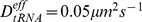. For discussion on uncertainties in 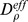, see Text S1.

In our previous analysis of tRNA diffusion during accommodation [49], we used Eq. 1 to calculate 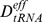, and compared it to the values obtained using a quasi-harmonic approximation, which accounts for the local curvature of each free-energy basin [54]. When using Eq. 1, the obtained diffusion coefficients were smaller (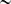
1/2) than those using the quasi-harmonic approximation [49]. One explanation for the attenuated values when using Eq. 1 is that the local curvature of the basin can reduce growth of 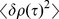 at long time. That is, as the system samples higher free-energy configurations, it eventually is thermodynamically favorable to return to the minimum. This can lead to decreased values of 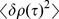 at large 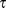, which would be associated with smaller values of 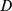. While it would be beneficial to directly compare the two approaches, in addition to other proposed methods [41]–[46], the current data set is not sufficient for such comparisons. Additionally, 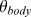 and 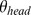 do not appear to fluctuate about a single well-defined energetic minimum (Fig. 4) during the course of the simulation, thus the quasi-harmonic approximation is not expected to provide reliable estimates for the present data set. Taken together, these considerations suggest that the diffusion coefficients reported here may be lower-bound estimates for the effective diffusion coefficients for 30S-body and 30S-head rotation.

### Effective diffusion, short-scale energetic roughness and short-time dynamics

Effective diffusion coefficients describe the short length-scale energetic roughness [1]–[3], allowing us to infer the energetic character of the landscape at different functional stages. The scale of the local energetic roughness 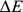 relates the effective diffusion and the diffusion of a free molecule, according to the relation:
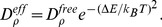
(2)We find that 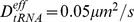 for the classical A/A-P/P-configured tRNAs, which is consistent with previously reported values [49]. However, it is significantly lower than for the A/T-configured tRNA molecule (i.e. the configuration in which aminoacyl-tRNA is delivered to the ribosome), where 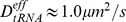 (nearly identical to the free diffusion in solution). This suggests that the energetic roughness increases as a tRNA molecule enters the ribosome and reaches a value of 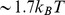
(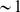 kcal/mol) as it maneuvers through the interior of the particle.

While we consider these values of the diffusion to be initial estimates, additional considerations suggest the presented values are reliable measures. First, the diffusive regime for each coordinate is reached at lag times (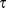) of 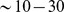 ns. Since this is far faster than the full-scale rotations implicated during translocation (milliseconds), these measures of 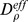 describe shorter-scale processes and should not be heavily influenced by large-scale barrier crossing processes. Second, in the course of the simulation, there does not appear to be a strongly-preferred orientation of the domains. This suggests that the energetic basin of attraction associated with the classical configuration is not sharply defined. As discussed above, the local curvature may have an impact on 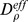. However, the absence of a well-defined minimum would suggest that this effect will be small. In other words, the diffusive time-regime is far shorter than the timescale associated with barrier-crossing attempts (see next section). Finally, the obtained diffusion coefficients do not differ significantly when only the first half, or second half, of the simulated data is used for analysis (Fig. S4), each of which samples different ranges of 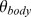 and 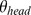. The two halves also provide similar values of 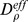, which would make it surprising if there is a strong coordinate-dependence in the vicinity of the classical configuration.

### Using the diffusion to connect the energy landscape and kinetics of rearrangements

**Figure 5:**
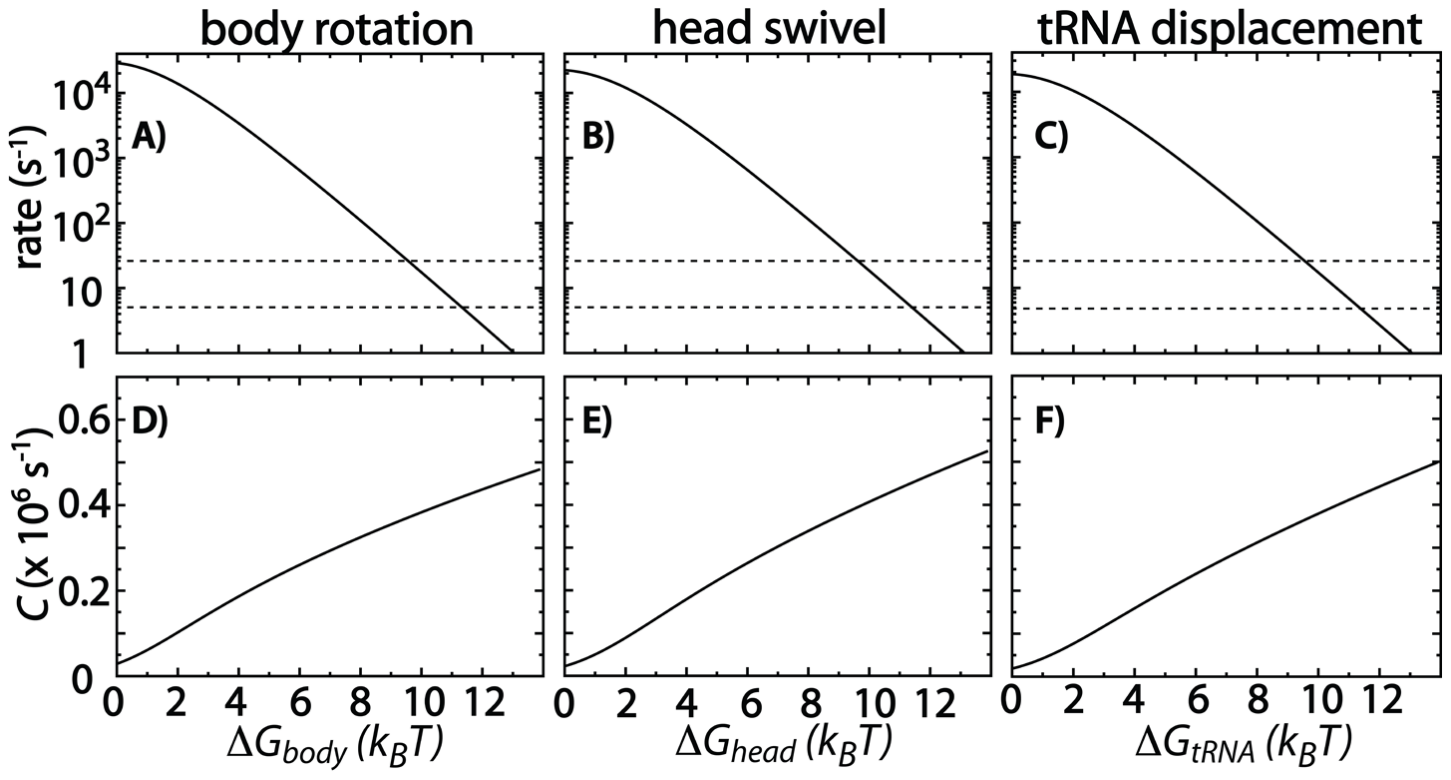
Rates, free-energy barriers and crossing attempt frequencies. Using 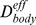, 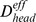, and 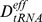, the rates of barrier crossing were calculated as functions of the barrier heights for A) body rotation, B) head swivel, and C) tRNA displacements. From the rates, the barrier-crossing attempt frequencies 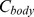, 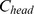, and 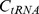 were derived (D–F). With these values, the energy landscape may be quantified for any kinetic scheme that can be decomposed into body rotation, head swivel and tRNA displacement. Dashed lines mark 5 and 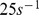 (range of rates measured for translocation [59]–[64]), providing an upper-limit range for the barrier height associated with each substep of translocation.

Since the presented analysis indicates that 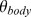, 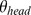 and 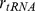 capture diffusive aspects of ribosome dynamics, we will use them to relate the free-energy barriers and kinetics associated with translocation-related structural rearrangements. The rates of body rotation, head-swiveling, and tRNA displacements are related to the underlying free energy according to [2], [3]:
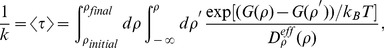
(3)where 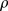 is 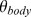, 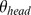 or 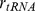. 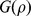 is the short-length scale averaged free energy as a function of each coordinate. 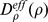 is the effective diffusion coefficient in 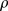-space. Here, we simplify the integral by treating 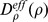 as a constant for each transition. While the diffusion may vary along each coordinate, the extent to which 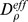 changes will be determined by the magnitude of the short-length scale roughness along each. Since the types of chemical interactions (i.e. protein-RNA, or RNA-RNA interactions) are similar during each rearrangement, including at the subunit bridges [21], it is not expected that the coordinate dependence will be large for subunit rotation. To emphasize this point, it is instructive to consider protein folding studies, where coordinate-dependent diffusion has been detected through a variety of computational and experimental methods [41]–[46], [55]–[58]. During protein folding, the polymer chain transits from an unfolded state, which is dominated by protein-solvent interactions, to a folded ensemble that is composed of protein-protein interaction. Despite this drastic change in the local chemical environment during folding, most studies have reported only modest changes in 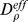 (factors of 2–5). In the present study of the ribosome, there are not large changes in the chemical composition of the subunit interfaces, making it reasonable to expect that the scale of the energetic roughness will not change significantly during each rearrangement. Nonetheless, since it is possible that there will be coordinate dependence, the presented estimates of 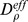 should be considered baseline estimates. As the coordinate-dependence of the diffusion is characterized, the presented calculations may be further refined to provide a more precise relationship between the free energy and the kinetics. With the calculated values of 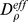, through numerical integration (See Methods) we calculated the rates of the rearrangements (body and head rotation and tRNA displacements) as functions of the barrier heights (Fig. 5A–C). The rate of translocation has been reported to be 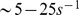, depending on the experimental conditions used [59]–[64]. By definition, each substep of translocation must be faster than the full process. Thus, our analysis indicates that the barriers for each substep are unlikely to exceed 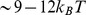 (Fig. 5).

Diffusion leads to free-energy barrier-crossing attempts and the barrier height determines the probability of successfully crossing [1]–[3], [49], [57], [65]. For a two-state transition (i.e. two energetic basins separated by a single barrier) the rate may be approximated in terms of an attempt frequency 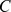 and free-energy barrier 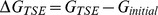: 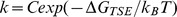, where 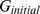 and 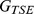 are the free energy of the initial ensemble and the TSE. 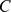 is process-specific. For example, biomolecular folding is associated with prefactors of 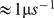 [1], [57], [66], while simulations suggest attempt frequencies for tRNA accommodation of 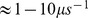 (Ref. [49]). Similarly, the calculated values of 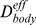 and 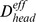 suggest that 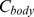 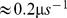 and 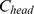 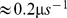 (Fig. 5, 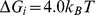 is used for comparison). For tRNA displacements that occur during hybrid-state formation and translocation, 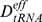 suggests 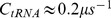. It is notable that the values of 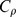 are comparable for all three processes, even though they take place in distinct coordinate spaces and have unique values of 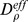. That is, the attempt frequencies are the result of both the magnitude of the diffusion, and the length scale of the rearrangement (Eq. 3). For these processes, the large rearrangements are accompanied by larger values for the diffusion, which leads to comparable attempt frequencies. In contrast, the attempt frequency for tRNA accommodation was found to be larger than for tRNA displacements associated with translocation. Since these processes occur on similar length-scales, this difference highlights the contribution of the diffusion coefficient to the attempt frequency. These similarities and differences between the attempt frequencies in each space demonstrate the balance between length scale, energetic roughness, diffusion and the free-energy barriers of conformational transitions in the ribosome.

## Discussion

The ability to rigorously interconvert between the energy landscape and kinetics will be essential in order to unambiguously quantify the features of the biomolecular landscapes that underpin function. With knowledge of the diffusive properties, theoretical and experimental probes of the energy landscape may be directly compared to kinetic measurements, which will enable a comprehensive picture of the landscape to emerge. Thus, the diffusion provides a unifying foundation for understanding and interpreting all available data for a given biological process. In the presented study, we have made the first steps towards establishing such a framework for tRNA translocation in the ribosome. To do this, we probed the diffusive characteristics of subunit rotations and tRNA displacements, essential sub-processes that facilitate protein elongation in the cell. As computer hardware continues to increase in power, and new computational algorithms and models are developed, the current study will provide the context for understanding a gamut of biophysical measurements and predictions. Of particular interest are the detailed features of the underlying energy landscape, as well as the robustness of ribosome dynamics to external perturbations. Similar to macroscopic machines, by understanding the interplay between the moving parts of these systems, it may be possible to design strategies to exploit this knowledge and provide precise regulation of biomolecular dynamics in the cell. In such efforts, the presented approach for bridging kinetics and free-energies provides a way to systematically verify predictions about the landscape against experimental data. These tools allow us to integrate complementary information from experimental and computational techniques, which will be crucial when identifying the features of the energy landscapes that govern biological function.

## Methods

### Simulation details

The simulation is a direct continuation of our previous 100 ns explicit-solvent simulation of the ribosome [67]. The simulation is based on the high resolution crystallographic structure of an *E. coli* ribosome in a pre-translocation (classical A/A-P/P) configuration (PDB ID: 2I2P and 2I2T. [68]). The simulation was performed on 2048 (on NMCAC Encanto) and 1024 (on TACC Lonestar) compute cores using Gromacs v4.5.3 [69], [70], with a peak performance of 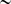
15 nanoseconds/day. The AMBER99p force field [71], [72] was employed. [KCl] = 100 mM and 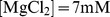, yielding 388 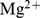, 6272 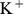 and 2831 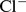 ions. 602587 SPC/E water molecules were included, for a total system size of 2070120 atoms. A 1.3 microsecond production run was performed with the Verlet integration scheme [73] and a 2 femtosecond time step. The NPT ensemble was sampled, where the system was coupled to a temperature bath of 300 K through use of the Nosé-Hoover thermostat [74], [75]. Pressure coupling was achieved through employment of the Berendsen algorithm, with a pressure of 1 bar, relaxation time of 2.5 picoseconds, and compressibility of 4.5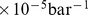 [76]. While the choice of thermostat and barostat could impact the observed kinetic properties, both operate by modulating average quantities. Due to the large number of atoms in this system, fluctuations in the average quantities (such as average kinetic energy per atom) will be relatively small, and the coupling baths should have only a marginal effect on the kinetics. Supporting this, previous simulations of the A/A-P/P configuration [49] that used an alternate thermal coupling algorithm provided effective diffusion coefficients for movement along 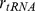 that were similar to the values reported here. Covalent bonds were constrained using the LINCS algorithm [77], while the cutoff distances for the van der Waals and Coulomb interactions were both chosen to be 0.9 nm. The long-range electrostatic interactions were treated by the PME algorithm [78], with a tolerance of 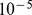 and an interpolation order of 4. Complete details on equilibration, structural modeling and the initial configuration may be found elsewhere [67].

### Identifying the core residues: An iterative-exclusion algorithm

During translocation by the ribosome, there are multiple large-scale rotary motions that facilitate tRNA movement (Fig. 2). To probe the collective rotary motions of the subunits, and exclude independent fluctuations of individual residues, we identified sets of core residues for the 50S subunit, 30S body and 30S head. In doing so, we only considered the rRNA portions of each region. These regions undergo sub-Å internal displacements, allowing their average orientations to be used to measure body and head rotation. To identify the core residues, we started with sets of candidate residues and then iteratively excluded highly-fluctuating ones, until a set was identified for which the rmsf is less than 1 Å for every residue. The following protocol was employed:

1. Start with a set of candidate residues for the 50S, 30S-body and 30S-head. Only rRNA residues were included in the candidate groups. Here, we refer to this set of residues as 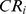, where i = 50S, 30S-body, or 30S-head. For 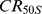, all 23S rRNA residues were considered viable candidates (N = 2903). For 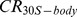, 1060 residues in the C, 5′ and 3′m regions were considered (U5-U920 and C1397-U1540). For 30S-head rotation, the residues in the head that are near the 50S-30S-tRNA interface (A935-G1047 and C1210-U1380) were considered candidates (N = 284).
2. Calculate the structural rmsf for all non-hydrogen atoms of each group 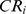. The g_rmsf module in Gromacs was used. For each calculation, the rmsf was calculated for the first 1000 ns of the simulation, sampling coordinates from every 1 ns of simulation. Structure alignment of the candidate residues was performed and the rmsf of each atom was calculated. The rmsf was then averaged by residue.
3. Remove residues from 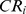 that exceed a threshold rmsf of 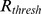. 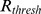 was initially set to 5 Å.
4. Iteratively calculate the rmsf and remove residues from 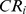 that have 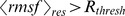 (steps 2 and 3), until the rmsf values of all residues in 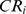 are below 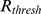.
5. Reduce the value of 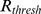 and repeat steps 2–4. 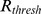 was sequentially reduced from 5 to 4, 3, 2, 1.5 and 1 Å.

Upon completion of this iterative-exclusion algorithm, there were 1353, 443 and 178 rRNA residues in the core groups of the 50S, 30S body and 30S head (Figs. 3B, 3C and S1; See Text S1 for list of core residues).

### Defining the rotation coordinates 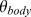 and 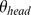


To define the coordinates for rotation ( 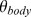 and 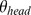 ), the core residue configurations were compared for classical and rotated configurations of the ribosome. First, for each structural model (classical, body-rotated, head-rotated), reference configurations of the core residues were spatially aligned to the 23S, 16S body and 16S head regions. This initial alignment provided an average orientation (i.e. the “idealized” coordinates) of each group, thereby ensuring that the rotation metrics probe the collective rotation of the groups and not the independent fluctuations of individual atom. Next, the idealized coordinates were compared for each structural model. Specifically, to define , all possible vectors that can be defined by two P atoms in 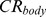 were calculated for the classical and body-rotated configuration. The atom-pair vector that undergoes the largest change in angle was then used to define the rotation plane for 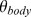. An analogous strategy was used to define 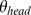. All scripts necessary to calculate these angles will be made available online, upon publication. For complete details, see Text S1.

### Calculating rates for substeps of translocation

With values of 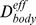, 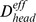, and 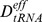 the rates of body rotation, head rotation, and tRNA displacements were calculated from Eq. 3. When 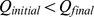, the lower bound on the inside integral is 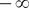, otherwise it is 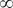. Consistent with previous studies [49], we numerically integrated Eq. 3 through the use of free-energy surfaces that have the functional form 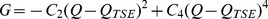 (Fig. S5), where 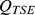 is the position of the free-energy barrier and 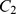 and 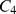 were set such that the endpoints are minima. Other functional forms were considered (Fig. S6), however, for sufficiently large barriers, the functional form does not have a sizable effect on the rate [65]. This point was also explicitly shown in Ref. [49]. Based on the analysis of cryo-EM and x-ray models (See Table S1), the bounds of integration corresponded to changes in each coordinate of 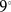 (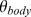), 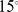 (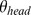) and 22 Å (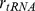). As discussed in the Results, the simulation was performed at 300 K (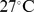), whereas many experiments are performed at 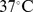. Accordingly, the diffusion coefficients presented here are slightly lower that what should be used when interpreting experiments at higher temperatures. For other kinetic models for which experimental data may become available, the limits of integration may be modified, thus providing a quantitative bridge between the landscape and kinetics for other kinetic representations of translocation.

As expected from the arguments of Kramers [3], [57], [65], for a substantial free-energy barrier (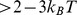) separating two free-energy minima, the mean first passage time may be approximated according to the relation:
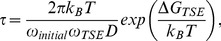
(4)where, 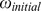 and 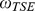 are the curvatures of the free-energy surface in the initial basin and the TSE, and 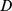 and 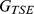 are the diffusion and free energy of the TSE. Accordingly, for any relatively smooth function that has a single, well-defined peak separating two fixed endpoints, the curvature of the basin and TSE will be within a relatively small range of values. In the present study, we employed a symmetric functional form for the barrier (Fig. S5), in order to calculate the relationship between the rates and the free-energy surface. Previously, we explicitly calculated the rates using alternate symmetric functional forms, and found the rates to be robust [49]. Here, we have additionally calculated the rates for asymmetric barriers (Fig. S6), which further highlights the robustness of the rates to the functional form. That is, for a barrier separating two basins of attraction by 15 distance units (e.g. 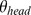 ), the calculated rates only vary by a factor of up to two when the free-energy peak is centered at 3, 7.5, or 12 distance units. Accordingly, for a given rate, the effect of this feature will only alter the predicted barrier height by a maximum of 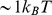.

## Supporting Information

Figure S1Definitions of core residues. (top) Candidate residues to define the core regions of the 23S (orange), 16S head (red) and 16S body (yellow). All 23S residues were considered candidates with which to define the 23S core. However, the 16S head and 16S body were manually partitioned and analyzed separately. (bottom) From the first 1 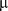
s of simulation, subsets of residues were identified within each group (23S, 16S head and 16S body) that had rmsf values that were less than 1 Å. “Mobile” residues (i.e. rmsf 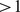 Å) are shown in gray (23S), or cyan (16S). Secondary structure image is from the Noller Laboratory website, and was recolored to depict the core groups identified here.(EPS)

Figure S2Structural fluctuations estimated from crystallographic refinement. rmsf values were obtained from PDB entry 3F1F, via the relation 
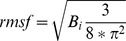
 [53]. Here, the rmsf values are averaged over all heavy atoms in each residue. Overall, the head region of the 16S rRNA has higher values than the body.(TIF)

Figure S3Displacement-squared as a function of lag time. (left) The displacement squared of 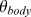 and 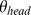 with linear fits to 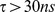 in gray. (right) Displacement squared for tRNA displacements with a linear fit for 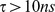 in gray.(TIF)

Figure S4Uncertainty in measures of the diffusion coefficients. From the 1.3 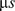 simulation, the average displacement-squared (as functions of lag time 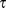) along each coordinate was calculated using the first and second 650 ns of the simulation. For each subset, 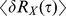 was fit to a linear function, in order to extract the slopes (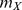), which are related to the diffusion coefficients according to the relation 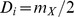. These fits yield values of 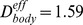 and 1.10 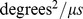, 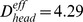 and 2.32 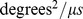, and 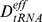 = 0.059 and 0.038 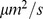. When using these values to obtain barrier heights for a given rate, these variations in 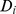 will lead to changes in the estimated barrier heights that are less than 1 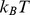, and they may therefore be considered relatively small uncertainties.(TIF)

Figure S5Functional form of 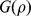 used for rate calculations. For all calculations of rates that use Equation 3, the following functional form of 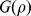 was used: For 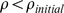, 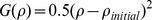. For 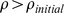, 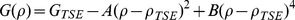, where 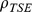 is the location of the TSE, 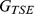 is the barrier height, and 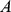 and 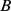 were set such that 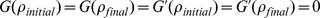. Since 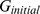 is defined to be 0, 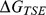 is equal to 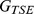. For a given calculation, the barrier height and the locations of the basins were adjusted to values appropriate for the process of interest (i.e. body rotation, head rotation, tRNA displacement). It was previously shown that the results are robust to the precise functional form [49].(TIF)

Figure S6Rates are robust to the functional form of the free-energy 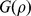. In the main text, the rates are reported for a symmetric functional form of 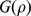 (red curve). When the location of the peak is varied, the rates for a given barrier only vary by approximately a factor of two. Accordingly, when determining barrier barrier height for given rate, the corresponding barrier height will be altered by less than 1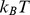.(TIF)

Figure S7Drift in 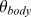 and 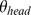 attenuates after 1 microsecond of simulation. After 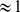 µs, both 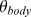 and 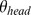 exhibit minimal drift over the final 300 ns of the simulation. Linear fits to each (light blue) have slopes of 0.06 and 0.4 degrees per microseconds for 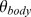 and 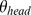.(TIF)

Figure S8Displacement-squared for alternate rotation coordinates. The displacement squared of 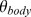 and 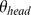 are shown in black. If the coordinates are not idealized (i.e. averaged) prior to calculating 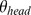 and 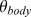, the coordinates have additional fluctuations that arise from the motions of individual atoms, and not the collective dynamics (red). Similarly, if all candidate residues are included in the averaging step, as opposed to only the core residues (green), then the coordinates have fluctuations that arise from structural rearrangements that are not due to collective rearrangements, such as fluctuations in the L1 and L11 stalks. For both 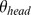 and 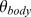 the fluctuations are smallest when idealization is performed for the core residues only. Additionally, the linear correlation coefficients (c.c.) are 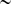 1.0 for the idealized-core curves, whereas c.c. is smaller for the other measures, suggesting that motion in those spaces is less diffusive.(TIF)

Table S1

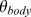
 and 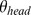 values for PDB-deposited structures. 1–19 are from *E. coli* and 20–26 are from *T. Thermophilus*. 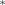
Reference configuration for the classical head and body. 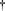
Reference for rotated body. 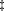
Reference for swiveled head. 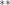 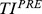 configuration and 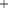 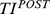 configuration described in Ref. [23].(PDF)

Text S1Supporting discussion and methodological details. Overview of elongation, details for 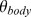 and 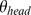 calculations, core group descriptions, temperature effects and uncertainty analysis.(PDF)
