# Supplementary figures and images for: Connecting the Kinetics and Energy Landscape of tRNA Translocation on the Ribosome
Source: PLoS Comput Biol. 2013 Mar 21;9(3):e1003003. doi: 10.1371/journal.pcbi.1003003 (PMC3605090; doi:10.1371/journal.pcbi.1003003)

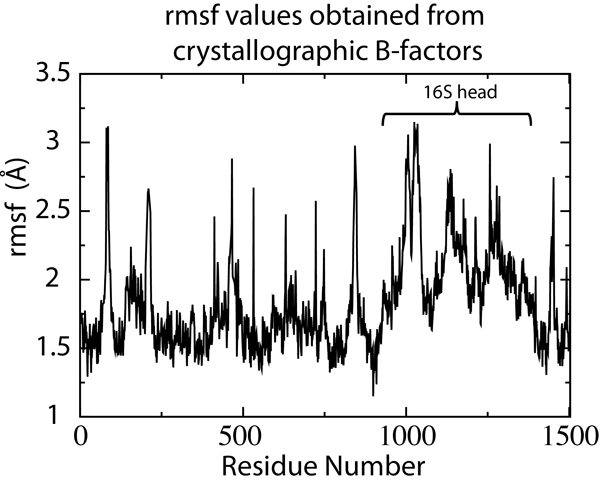

Supplement: Figure S2 — Structural fluctuations estimated from crystallographic refinement. rmsf values were obtained from PDB entry 3F1F, via the relation [53]. Here, the rmsf values are averaged over all heavy atoms in each residue. Overall, the head region of the 16S rRNA has higher values than the body. (TIF) [file pcbi.1003003.s002.tif]

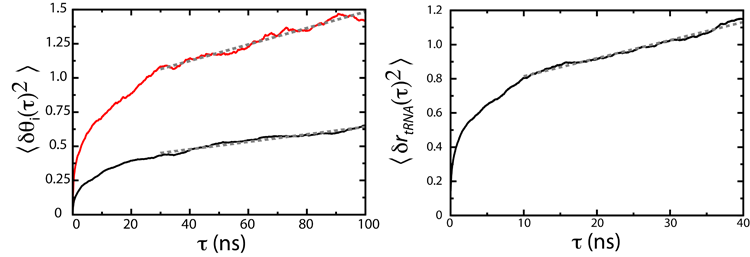

Supplement: Figure S3 — Displacement-squared as a function of lag time. (left) The displacement squared of and with linear fits to in gray. (right) Displacement squared for tRNA displacements with a linear fit for in gray. (TIF) [file pcbi.1003003.s003.tif]

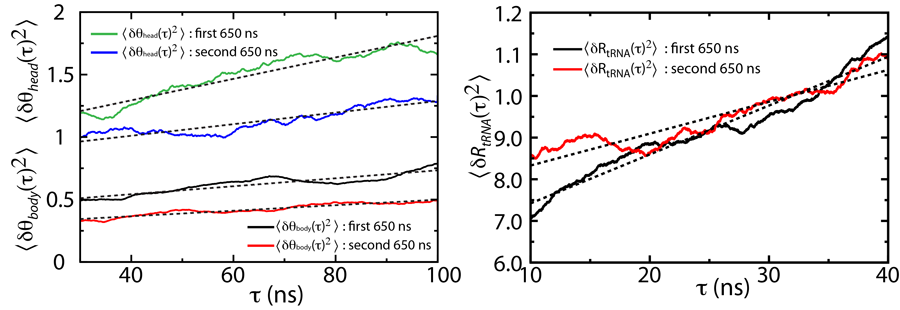

Supplement: Figure S4 — Uncertainty in measures of the diffusion coefficients. From the 1.3 simulation, the average displacement-squared (as functions of lag time ) along each coordinate was calculated using the first and second 650 ns of the simulation. For each subset, was fit to a linear function, in order to extract the slopes (), which are related to the diffusion coefficients according to the relation . These fits yield values of and 1.10 , and 2.32 , and = 0.059 and 0.038 . When using these values to obtain barrier heights for a given rate, these variations in will lead to changes in the estimated barrier heights that are less than 1 , and they may therefore be considered relatively small uncertainties. (TIF) [file pcbi.1003003.s004.tif]

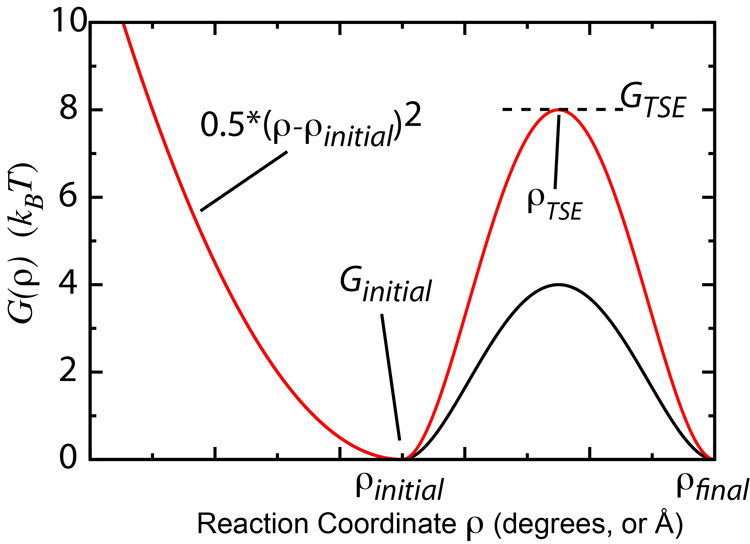

Supplement: Figure S5 — Functional form of used for rate calculations. For all calculations of rates that use Equation 3, the following functional form of was used: For , . For , , where is the location of the TSE, is the barrier height, and and were set such that . Since is defined to be 0, is equal to . For a given calculation, the barrier height and the locations of the basins were adjusted to values appropriate for the process of interest (i.e. body rotation, head rotation, tRNA displacement). It was previously shown that the results are robust to the precise functional form [49]. (TIF) [file pcbi.1003003.s005.tif]

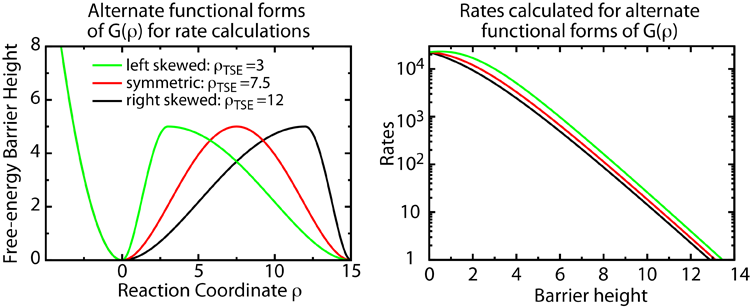

Supplement: Figure S6 — Rates are robust to the functional form of the free-energy . In the main text, the rates are reported for a symmetric functional form of (red curve). When the location of the peak is varied, the rates for a given barrier only vary by approximately a factor of two. Accordingly, when determining barrier barrier height for given rate, the corresponding barrier height will be altered by less than 1. (TIF) [file pcbi.1003003.s006.tif]

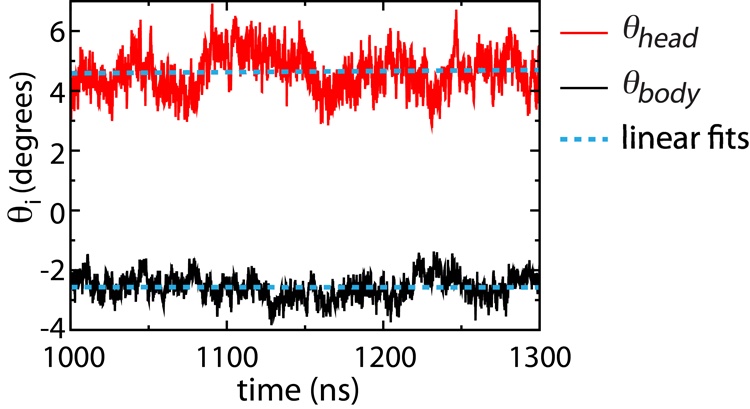

Supplement: Figure S7 — Drift in and attenuates after 1 microsecond of simulation. After µs, both and exhibit minimal drift over the final 300 ns of the simulation. Linear fits to each (light blue) have slopes of 0.06 and 0.4 degrees per microseconds for and . (TIF) [file pcbi.1003003.s007.tif]

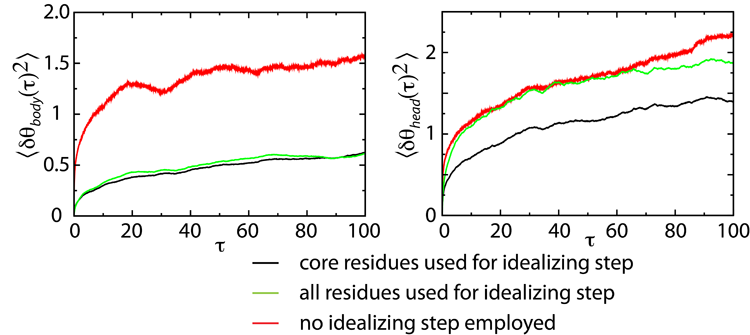

Supplement: Figure S8 — Displacement-squared for alternate rotation coordinates. The displacement squared of and are shown in black. If the coordinates are not idealized (i.e. averaged) prior to calculating and , the coordinates have additional fluctuations that arise from the motions of individual atoms, and not the collective dynamics (red). Similarly, if all candidate residues are included in the averaging step, as opposed to only the core residues (green), then the coordinates have fluctuations that arise from structural rearrangements that are not due to collective rearrangements, such as fluctuations in the L1 and L11 stalks. For both and the fluctuations are smallest when idealization is performed for the core residues only. Additionally, the linear correlation coefficients (c.c.) are 1.0 for the idealized-core curves, whereas c.c. is smaller for the other measures, suggesting that motion in those spaces is less diffusive. (TIF) [file pcbi.1003003.s008.tif]
