# Supplementary material for: Connecting the Kinetics and Energy Landscape of tRNA Translocation on the Ribosome
Source: PLoS Comput Biol. 2013 Mar 21;9(3):e1003003. doi: 10.1371/journal.pcbi.1003003 (PMC3605090; doi:10.1371/journal.pcbi.1003003)
Supplement: Table S1 — and values for PDB-deposited structures. 1–19 are from E. coli and 20–26 are from T. Thermophilus. Reference configuration for the classical head and body. Reference for rotated body. Reference for swiveled head. configuration and configuration described in Ref. [23]. (PDF) [file pcbi.1003003.s009.pdf]

	PDB	θ_{body}	θ_{head}
1	3R8O*	0.0	0.0
2	3R8N†	9.1	3.4
3	2AW7‡	-1.2	15.3
4	2AVY	-2.3	6.5
5	3I1M	5.7	10.0
6	3I1O	-0.2	6.9
7	3I1Q	5.7	0.1
8	3I1S	-1.6	2.9
9	3I1Z	6.0	0.0
10	3I21	-0.4	1.8
11	1PNS	-1.7	1.3
12	1PNX	-1.2	1.0
13	1VS5	-2.3	6.5
14	1VS7	-1.3	15.3
15	2QBD	-2.4	6.6
16	2QBF	-1.2	15
17	3DF1	-2.3	6.5
18	3DF3	-1.2	14.9
19	2I2P	-1.7	3.1
20	2J00	0.8	-1.3
21	2XQD	0.8	-1.0
22	2XSY**	6.6	4.3
23	2XUY+	3.3	18.7
24	2Y0U	0.0	0.7
25	3I8G	0.2	0.0
26	3MR8	-0.8	1.3
