# Supplementary material for: Connecting the Kinetics and Energy Landscape of tRNA Translocation on the Ribosome
Source: PLoS Comput Biol. 2013 Mar 21;9(3):e1003003. doi: 10.1371/journal.pcbi.1003003 (PMC3605090; doi:10.1371/journal.pcbi.1003003)
Supplement: Text S1 — Supporting discussion and methodological details. Overview of elongation, details for and calculations, core group descriptions, temperature effects and uncertainty analysis. (PDF) [file pcbi.1003003.s010.pdf]

Supporting Information: Connecting the kinetics and energy landscape of tRNA translocation on the ribosome

Paul C. Whitford, Scott C. Blanchard,
Jamie H. D. Cate, Karissa Y. Sanbonmatsu

Contents

1 Supplementary Discussion **2**

1.1 Overview of elongation, translocation and subunit rotations 2

2 SI Methods **5**

2.1 Defining the rotation coordinates θ_{body} and θ_{head} 5

2.1.1 Defining and calculating θ_{body} 6

2.1.2 Defining and calculating θ_{head} 8

2.2 Alternate criteria for defining core residues 9

3 SI Results **10**

3.1 Residue IDs used to define the core residues (E. coli numbering): 10

3.2 Temperature Dependence of D 12

3.3 Uncertainty in the estimates of the diffusion coefficients 13

1 Supplementary Discussion

1.1 Overview of elongation, translocation and subunit rotations

During the elongation cycle of translation, large-scale rearrangements in the ribosome (Fig. 2) allow transfer RNA (tRNA) molecules to transiently associate and decode messenger RNA (mRNA). When adding an amino acid to the growing protein chain, each tRNA molecule transits across the full length of the ribosome ($\approx$200 Å). This process is orchestrated by over 70 gene products, for which structural methods (x-ray crystallography and cryo-EM) have produced snapshots of the moving parts at various stages of function (*1–6*). Kinetic measurements have elucidated the rates of structural rearrangements (*7, 8*), while time traces of individual complexes are accessible from single-molecule FRET (smFRET) experiments (*9, 10*).

The ribosome possesses three tRNA binding sites that span the 30S and 50S subunits: the A, P and E sites. After aminoacyl-tRNA enters the A site, the incoming amino acid is added to the growing protein chain, which is attached to the P-site tRNA. In order for elongation to continue, the A-site tRNA must move to the P site and the P-site tRNA moves to the E site, a rearrangement known as translocation. Prior to translocation, the tRNA molecules are described as being in the A/A-P/P configuration, and after translocation they adopt the P/P-E/E configuration (Fig. 2). The convention for naming tRNA configurations is the following: A/B-C/D, where A and B indicate the binding sites on the small and large subunit that the first tRNA is associated with, and C and D indicate the sites that second tRNA is associated with. For example, A/A-P/P indicates that one tRNA is associated with the A sites of both subunits and the second tRNA is associated with the P sites of both subunits. In contrast, A/A-P/E would indicate that one tRNA is associated with both A sites, while the second tRNA is associated with the P site of the small subunit and the E site of the large subunit (a so-called "hybrid" configuration).

While the precise details of the timing and degree of coupling between each translocation-related motion is still debated, it is well established that during tRNA translocation, there are at least two large-scale rotary motions that involve the 30S subunit: 30S-body rotation (Fig. 3D) and 30S-head swivel (Fig. 3E). The relative positioning of the body and head has been shown to vary in a number of x-ray crystallographic models and cryogenic-electron microscopy reconstructions (Table S1) (*1, 4, 6, 11–23*). Further, the presence and interconversion between these conformations has been observed in FRET measurements (*24–26*). In addition to rotations of the subunits, populations of intermediate hybrid-state tRNA configurations have also been detected with biochemical, single-molecule and x-ray methods (*17, 25, 27–34*). As described above, the intermediate hybrid configurations are characterized by each tRNA bridging two different sites on the subunits, such as the A site of the small subunit and P site of the large subunit.

While there is overwhelming evidence demonstrating that subunit rotation is correlated with tRNA translocation, and that the transition between the A/A-P/P and P/P-E/E configurations is not two-state-like (i.e. there is not a single free energy barrier), there is still significant controversy regarding the the exact sequence and scale of each motion, as well as the degree of coupling between rotation and tRNA displacements. Accordingly, here, we focus on providing a general set of tools for analyzing any kinetic/structural representation that decompose translocation into body rotation, head swiveling and tRNA displacements. To do so, we have calculated the diffusion coefficients in each coordinate space and provide demonstrative calculations that illustrate how the diffusion may be used for one specific description of the rearrangements. Specifically, we calculate the rates according to the following sequence of conformations, which are based on recent atomic models obtained from cryo-EM studies (*35*):

1. Classical: A/A-P/P tRNA. θ_{body}=0°, θ_{head}=0°, Δr_{tRNA}=0Å (Δr_{tRNA} is the difference in distance from that found in the A/A-P/P configuration)

2. Body rotated: A/A-P/P. θ_{body}=9°, θ_{head}=0°, Δr_{tRNA}=0Å

3. P/E hybrid formed: A/A-P/E. θ_{body}=9°, θ_{head}=0°, Δr_{tRNA}=22Å

4. Head rotated: A/A-P/E. θ_{body}=9°, θ_{head}=15°, Δr_{tRNA}=22Å

5. A/P hybrid formed: A/P-P/E. θ_{body}=9°, θ_{head}=15°, Δr_{tRNA}=0Å

6. Relax to Classical: A/A-P/P tRNA. θ_{body}=0°, θ_{head}=0°, Δr_{tRNA}=0Å

For any other experimental result (e.g. a specific number of configurations associated with translocation), the rate-barrier calculations presented here may be revised to describe the specific sequence of events observed in a given experiment. For example, while a recent experimental study used a similar kinetic scheme to interpret bulk FRET measurements (*?*), the values of the endpoint positions associated with each substep could be easily modified based on other emerging experimental data. The diffusion coefficients reported here would then be used to calculated the barrier-rate relationship for those results, and the observed rates of each conformational event could be used to infer the scale of the associated barrier. Additionally, as evidence is provided for the degree of coupling between multiple motions, such as head-swivel and A/P formation, then multi-dimensional energy surfaces may be used, along with the values of D_ρ reported here, to relate the kinetics and thermodynamics of those data sets.

While evidence is mounting that subunit rotation reduces the free-energy barriers of tRNA translocation, and that Elongation Factor-G (EF-G) facilitates rotation, tRNA molecules can also undergo spontaneous translocation, in both the forward (*37*) and reverse (*22*) directions. It is generally thought that the primary mode by which EF-G accelerates translocation is by shifting the free-energy landscape in the forward direction, or by reducing the large-scale free-energy barriers. If either of these modes is utilized by EF-G, then the effective diffusion coefficients presented here may be used to interpret translocation dynamics, with or without EF-G present.

That is, here, we report the effective (i.e. short length-scale averaged) diffusion. If the character of the short-scale roughness if not significantly altered by EF-G, but rather, the large-scale shape of the free-energy is modified by EF-G, then the effective diffusion coefficients should be similar between EF-G-free and EF-G-present systems.

2 SI Methods

2.1 Defining the rotation coordinates θ_{body} and θ_{head}

To define the coordinates for rotation (θ_{body} and θ_{head}), the core residue configurations were compared for classical and rotated configurations of the ribosome. First, for each structural model (classical, body-rotated, head-rotated), reference configurations of the core residues were spatially aligned to the 23S, 30S body and 30S head regions. This initial alignment provided an average orientation (i.e. the "idealized" coordinates) of each group, thereby ensuring that the rotation metrics probe the collective rotation of the groups and not the independent fluctuations of individual atom. Next, the idealized coordinates were compared for each structural model. Specifically, to define θ_{body}, all possible vectors that can be defined by two P atoms in CR_{body} were calculated for the classical and body-rotated configuration. The atom-pair vector that has the largest difference in angle was then used to define the rotation plane for θ_{body}. An analogous strategy was used to define θ_{head}.

By comparing the orientations of the core residues in x-ray and cryo-EM models, collective reaction coordinates were defined that describe the rotation of the 30S body and head (θ_{head} and θ_{body}: Fig. 3D and 3E). While other reaction coordinates were considered, including the RMSD from the starting-point and endpoint configurations, an appropriate coordinate must distinguish between the endpoints and the transition state ensemble (TSE). Further, similar values of the coordinate should correspond to structurally-similar configurations. Since RMSD is a spatially-isotropic descriptor, atomic displacements in any direction yield similar values.

For example, RMSD from the initial configuration (in this case, the classical configuration) can not distinguish between rotation in the (+) (i.e. towards a rotated/swiveled configuration) and (-) directions. Finite temperatures lead to constant structural fluctuations, where many are not in functional directions.

We also considered using standardized methods for probing collective rearrangements, such as Principal Component Analysis. However, these methods often require that the system fluctuate about a particular energetic minimum, in order to utilize a harmonic approximation to the fluctuations. As described in the main text, in our simulation, the system did not appear to sample a single energetic basin, but rather searched the θ_{body} and θ_{head} spaces for nearly 1 microsecond before relaxing into what may be a local energetic minimum (Fig. S7). Accordingly, the low-frequency PCA modes are not likely to be informative, for this particular simulation. Therefore, RMSD and PCA were not employed and θ_{body} and θ_{head} were constructed such that they only change values when the ribosome undergoes the collective rotary motions that have been implicated in translocation (*5, 6, 10, 21, 32*).

2.1.1 Defining and calculating θ_{body}

To determine the vector of rotation that defines θ_{body}, we utilized the definitions of core residues, in conjunction with structural models from x-ray crystallography and cryo-EM. To define θ_{body}, the following protocol was employed:

1. Align the models: Using VMD (*38*), we aligned the classical (PDB: 3R8O, 3R8T) and rotated (PDB: 3R8N, 3R8S) atomic models to a consistent reference frame through rmsd minimization of the P atoms of the 50S core residues (CR_{50S}).

2. Idealize the coordinates: A reference model of the core residues was fit to atomic models of the classical and 30S body-rotated configurations. The idealization step allowed for an average orientation of the group to be calculated, and it therefore removes the effects of

independent fluctuations of individual atoms on the rotation coordinate. For example, in Fig. S8, the displacement squared possesses more visible noise when idealization is not performed.

3. Prepare candidate vectors between atom pairs: For each model k (=classical, rotate), we calculated the normalized vectors that are defined by the positions of P-P atom pairs ij : $\mathbf{R}_{ij}^k = \frac{\mathbf{r}_i^k - \mathbf{r}_j^k}{|\mathbf{r}_i^k - \mathbf{r}_j^k|}$, where $\mathbf{r}_i^k$ is the position of the P atom in residue i.

4. Find the maximum angular difference between classical and rotated configuration: Identify the atom pair ij [1] that minimizes $d_{ij} = \mathbf{R}_{ij}^{classical} \cdot \mathbf{R}_{ij}^{rotated}$, where $d_{ij} = cos(\theta)$ and θ is the angle formed by $\mathbf{R}_{ij}^{classical}$ and $\mathbf{R}_{ij}^{rotated}$. Thus, minimizing d_{ij} is equivalent to maximizing the angle between the vectors. The ij pair that had a maximal angular difference between the classical and rotated body was G237:C811. The angle associated with this atom pair was 9.1° between the classical and rotated body configurations. These results were robust to the precise atom pair used, so long as the angle between the configurations is near maximal. Specifically, of the 66603 atom pairs considered, 13698 have an angular difference that is greater than 9°.

5. Define the rotation vector for θ_{body}: We defined the rotation vector $\mathbf{V}^{body}$, such that it is orthogonal to both $\mathbf{R}_{ij}^{classical}$ and $\mathbf{R}_{ij}^{rotated}$. θ_{body} is defined as rotation about the vector $\mathbf{V}^{body} = \mathbf{R}_{ij}^{classical} \times \mathbf{R}_{ij}^{rotated}$. $\theta_{body} = 0$ was defined in the direction of $\mathbf{R}_{ij}^{classical}$. The rotation vector $\mathbf{V}^{body}$ is depicted in Fig. 3D. If $\mathbf{V}^{body}$ were assigned based on the alternate 13697 atom pairs (see previous step), its direction would be nearly identical to that obtained for the employed atom pair ($\mathbf{V}_{used}^{body} \cdot \mathbf{V}_{alternate}^{body} > 0.989$).

[1] Atoms pairs were not considered if they were less than ~ 40 Å apart.

Using the trjconv[2] module of Gromacs, each frame of the simulated trajectory (every 10 ps) was aligned, based on the 50S core residues (CR_{50S}) (same reference structure as used in step 1). Next, the coordinates of the 30S-body core residues ($CR_{30S-body}$) were idealized (step 2). The vectors and angles were calculated for each frame using a combination of Gromacs modules and in-house scripts. θ_{body} was then calculated for each idealized coordinate set.

2.1.2 Defining and calculating θ_{head}

θ_{head} was defined according to an identical strategy as θ_{body}. For completeness, we provide the details explicitly. To determine the vector of rotation that defines θ_{head}, which measures head rotation, we utilized the definitions of core residues, in conjunction with structural models from x-ray crystallography and cryo-EM. To define θ_{head}, the following protocol was employed:

1. Align the models: Align the classical (PDB: 3R8O, 3R8T) and swiveled (PDB: 2AW7,2AWB) atomic models to a consistent reference frame through rmsd minimization of the P atoms in the 30S-body core residues ($CR_{30S-body}$).

2. Idealize the coordinates: Fit a reference model of the core head residues to atomic models of the classical and 30S head-swiveled configurations.

3. Prepare candidate vectors between atom pairs: For each model k (=classical, swiveled), calculate the normalized vectors defined by P-P atom pairs ij : $\mathbf{R}_{ij}^{k} = \frac{\mathbf{r}_i^k - \mathbf{r}_j^k}{|\mathbf{r}_i^k - \mathbf{r}_j^k|}$, where $\mathbf{r}_i^k$ is the position of the P atom in residue i.

4. Find the maximum angular difference between classical and swiveled configurations: Identify the atom pair ij [3] that minimizes $d_{ij} = \mathbf{R}_{ij}^{classical} \cdot \mathbf{R}_{ij}^{swiveled}$, where $d_{ij} = cos(\theta)$

[2] An unmodified version of trjconv will work, however, a few minor modifications were introduced that increased the performance significantly, for this particular calculation.

[3] Atoms pairs were not considered if they were less than $\sim$ 50 Å apart.

and θ is the angle formed by $\mathbf{R}_{ij}^{classical}$ and $\mathbf{R}_{ij}^{swiveled}$. Minimizing d_{ij} is equivalent to maximizing the angle between the vectors. The ij pair that had a maximal angular difference between the classical and swiveled head was A977:A1374. The angle associated with this coordinate was 15.3 ° between the classical and swiveled head reference configurations (Table S1). Consistent with the evaluation of θ_{body}, the results were robust to the precise atom pair used. Specifically, of the 5930 atom pairs considered, 1633 atoms pairs have an angular difference that is greater than 15°.

5. Define the rotation vector for θ_{head}: We defined the rotation vector $\mathbf{V}^{head}$, such that it is orthogonal to both $\mathbf{R}_{ij}^{classical}$ and $\mathbf{R}_{ij}^{swiveled}$. θ_{head} is defined as rotation about the vector $\mathbf{V}^{head} = \mathbf{R}_{ij}^{classical} \times \mathbf{R}_{ij}^{swiveled}$. θ_{body}= 0 corresponds to the direction of $\mathbf{R}_{ij}^{classical}$. The rotation vector $\mathbf{V}^{head}$ is depicted in Fig. 3E. If $\mathbf{V}^{head}$ were assigned based on the alternate 1219 atom pairs (see previous step), it would be nearly identical to that obtained for the employed atom pair, with $\mathbf{V}_{used}^{head} \cdot \mathbf{V}_{alternate}^{head} > 0.940$.

For each frame of the simulated trajectory (every 10 ps), the 30S-body core residues ($CR_{30S-body}$) were aligned to the same reference structure as used in step 1. Next, the coordinates of the head core residues ($CR_{30S-head}$) were idealized (step 2). Then, θ_{head} was calculated for each idealized coordinate set.

2.2 Alternate criteria for defining core residues

To determine the core residues, an alternate criterion that we chose not to employ was calculating the rmsf of all candidate residues and then keeping all that are below 1Å. This would result in a smaller number of atoms being included in each core group. With that approach, some relatively immobile residues will have an rmsf that is above the threshold of 1 Å and would therefore be excluded. For example, 1155 residues have a rmsf of less than 1 Å when the rmsf is calculated using all atoms in the 23S rRNA. In contrast, with the iterative-exclusion strategy,

1353 residues were identified that have an internal rmsf of less than 1Å. We did not employ a simple cutoff here, since we wanted to include all atoms that undergo the collective rotary motions.

Using a larger number of residues to define CR_X also reduces the contribution that random fluctuations of individual residues make to metrics that probe the average rotation of that group of atoms. Simple arguments can demonstrate this point. Thermal energy leads to random fluctuations of each residue in the (+) and (-) directions of any given rotation coordinate. For example, let us define an average measure of rotation as $ROT = \sum_i^N S_i/N$, where S_i describes the position of each atom along the rotation coordinate. If $\langle S_i \rangle = 0$, then $\langle ROT \rangle = 0$. However, the dispersion in ROT will not be zero. Rather, $\langle ROT^2 \rangle = \langle (\sum_i^N S_i/N)^2 \rangle$. If the atomic fluctuations are independent, then $\langle S_i S_j \rangle = 0$ for $i \neq j$ and $\langle ROT^2 \rangle = \frac{1}{N^2} \sum_i^N \langle S_i^2 \rangle$. If we assume $\langle S_i^2 \rangle \approx C$, where C is a constant, then $\langle ROT^2 \rangle = \frac{1}{N^2} CN = C/N$. Thus, the dispersion in ROT will scale with $1/N$. Accordingly, by increasing the number of atoms used to measure the average rotation of CR_X, we reduce the influence of random fluctuations of individual residues.

3 SI Results

3.1 Residue IDs used to define the core residues (E. coli numbering):

After applying the iterative-exclusive algorithm, the following residues were identified as core residues:

23S (N=1353): 13-20, 26-29, 31-31, 35-38, 45-49, 51-59, 67-75, 114-124, 126-130, 177-179, 182-184, 186-199, 201-212, 214-215, 219-255, 258-263, 266-266, 371-386, 391-402, 407-423, 427-430, 433-433, 442-454, 456-471, 474-476, 478-479, 511-527, 530-541, 553-562, 564-571, 573-575, 577-577, 579-585, 587-588, 598-612, 615-641, 647-651, 655-664, 668-670, 672-686, 690-699, 703-707, 725-731, 733-736, 741-741, 743-748, 753-754, 756-757, 759-775, 778-

783, 786-787, 791-792, 795-807, 809-835, 838-840, 856-858, 860-864, 909-913, 916-918, 939-948, 950-951, 953-957, 959-973, 976-981, 985-985, 991-1004, 1007-1008, 1010-1010, 1022-1022, 1029-1029, 1124-1129, 1131-1132, 1136-1143, 1152-1165, 1185-1203, 1213-1219, 1222-1228, 1233-1235, 1238-1239, 1244-1261, 1263-1268, 1270-1270, 1275-1275, 1277-1282, 1286-1290, 1292-1292, 1295-1299, 1301-1301, 1305-1310, 1312-1318, 1323-1323, 1326-1326, 1333-1339, 1341-1358, 1361-1365, 1369-1370, 1375-1382, 1384-1384, 1386-1389, 1393-1395, 1397-1399, 1404-1406, 1424-1433, 1469-1470, 1568-1574, 1597-1606, 1611-1614, 1617-1617, 1619-1624, 1628-1629, 1634-1634, 1637-1641, 1643-1643, 1648-1654, 1656-1659, 1661-1676, 1678-1678, 1682-1682, 1687-1687, 1691-1693, 1695-1698, 1700-1703, 1707-1708, 1755-1756, 1765-1766, 1769-1779, 1782-1791, 1795-1800, 1802-1807, 1809-1815, 1817-1825, 1828-1829, 1833-1834, 1842-1843, 1853-1854, 1862-1864, 1880-1882, 1890-1890, 1898-1899, 1901-1904, 1932-1938, 1940-1940, 1943-1948, 1953-1953, 1955-1955, 1958-1961, 1966-1970, 1972-1972, 1978-1981, 1983-1996, 1998-2010, 2012-2015, 2017-2025, 2027-2040, 2042-2057, 2060-2061, 2063-2075, 2078-2092, 2197-2197, 2224-2225, 2227-2268, 2270-2270, 2272-2278, 2280-2282, 2285-2291, 2325-2332, 2335-2335, 2337-2337, 2345-2351, 2354-2370, 2382-2383, 2385-2388, 2391-2395, 2399-2400, 2403-2405, 2407-2411, 2413-2421, 2424-2433, 2435-2439, 2441-2458, 2461-2468, 2482-2491, 2494-2504, 2507-2524, 2540-2546, 2548-2552, 2554-2554, 2557-2560, 2562-2564, 2566-2570, 2572-2583, 2588-2593, 2598-2599, 2603-2604, 2608-2610, 2612-2627, 2643-2643, 2646-2649, 2679-2681, 2683-2688, 2693-2701, 2708-2711, 2713-2715, 2718-2721, 2724-2725, 2727-2730, 2740-2741, 2765-2765, 2776-2779, 2781-2781, 2818-2819, 2822-2830, 2835-2842, 2876-2877

16S head (N=178): 935-951, 956-960, 962-964, 966-969, 971-971, 975-982, 984-990, 994-995, 1013-1017, 1046-1047, 1210-1210, 1215-1226, 1231-1241, 1249-1255, 1258-1261, 1268-1271, 1276-1277, 1279-1279, 1281-1285, 1287-1289, 1300-1337, 1340-1361, 1363-1363, 1365-1377

16S body (N=443): 7-12, 15-30, 32-43, 46-49, 51-59, 110-118, 123-129, 233-238, 243-243, 253-253, 276-279, 281-282, 288-305, 308-317, 320-324, 327-327, 329-330, 332-333, 350-358, 360-365, 370-370, 375-379, 386-397, 401-410, 417-418, 426-431, 436-440, 483-483, 495-496, 498-507, 509-530, 533-533, 535-563, 566-575, 577-590, 598-598, 606-608, 616-617, 622-623, 626-627, 644-645, 651-652, 654-656, 665-671, 724-733, 738-742, 751-775, 791-791, 802-834, 853-888, 900-901, 910-920, 1397-1397, 1399-1405, 1496-1513, 1522-1531

3.2 Temperature Dependence of D

The short-scale roughness may be used to extrapolate the temperature dependence of the effective diffusion coefficients, thus allowing the values reported here to be used for a range of comparisons between theory and experiments. The presented simulation was performed at 300 K, whereas experimental measurements are obtained at T_{exp} =310 K. Since a phase transition is not known to occur within this temperature range, $D_\rho^{eff}(T)$ (the effective diffusion at temperature T) may be estimated through the use of a first-order Taylor expansion of Eq. 2, about $T = T_0$:

$$\begin{aligned} D_\rho^{eff}(T) &\approx D_\rho^{free} e^{-(\Delta E/k_B T_0)^2} + 2 D_\rho^{free} e^{-(\Delta E/k_B T_0)^2} \left(\frac{\Delta E}{k_B}\right)^2 \frac{T - T_0}{T_0^3} \\ &= D_\rho^{eff}(T_0) \left(1 + 2\left(\frac{\Delta E}{k_B T_0}\right)^2 \frac{T - T_0}{T_0}\right). \end{aligned} \tag{S1}$$

According to this relation, for $T_0 = 300$ K and $T = T_{exp} = 310$ K, $D_\rho^{eff}(T_{exp})$ is not likely to be significantly larger than $D_\rho^{eff}(T_0)$. For $\Delta E \sim k_B T_0$ (as is the case for tRNA diffusion), $D_\rho^{eff}(310K) \approx 1.1 D_\rho^{eff}(300K)$. Even if ΔE were large ($\sim 5 k_B T_0$), $D_\rho^{eff}(T_{exp})$ would only increase by a factor of ~ 2, indicating that large changes in D_ρ^{eff} are not expected across this temperature range. Therefore, the variations in temperature are unlikely to increase the diffusion coefficient by more than a factor of 2. Since the barriers are logarithmically related to the rates,

for a given rate, temperature effects introduce an uncertainty in the barrier estimates of $< 1k_BT$. Consistent with physical intuition, since ΔE must be positive, the rates implicated for a specific barrier should be considered lower-bound estimates, when using $D_\rho^{eff}(T_0)$ to calculate rates at higher temperatures.

3.3 Uncertainty in the estimates of the diffusion coefficients

For the 1.3 μs simulation, all diffusion coefficients were calculated for subsets of the data. Specifically, for each coordinate, $\langle \delta X(\tau)^2 \rangle$ was calculated independently for the first 650 ns and the second 650 ns of the simulation (Fig. S4). The obtained D_X values varied by a factor of 1.4 to 2. When using diffusion coefficients to determine the barrier height, given a specific rate, this degree of uncertainty corresponds to an uncertainty in the barrier of less than $1k_BT$. For the rotation coordinates, $\langle \delta\theta_X(\tau)^2 \rangle$ becomes linear after $\sim$ 30ns. Since the linear portion is what the diffusion coefficient is extracted from, and it is necessary that the analyzed trace is at least 10-20 times longer than that, trajectories that are less than $\sim$600 ns will provide estimates that are extremely noisy. Ideally, one would have a trajectory that is hundreds of times longer than the local relaxation, but using one that is 20-50 times the scale of the relaxations, as done here, can provide reasonable initial estimates. Accordingly, here, we do not attempt to estimate the diffusion from shorter segments from the run, or from earlier simulations that were only 200-300 ns in duration (*39*).

References

1. T M Schmeing and V Ramakrishnan. What recent ribosome structures have revealed about the mechanism of translation. *Nature*, 461(7268):1234–42, Oct 2009.

2. A Yonath. Large facilities and the evolving ribosome, the cellular machine for genetic-code translation. *J Royal Soc Inter*, 6:S575–S585, Aug 2009.

3. N Demeshkina, L Jenner, E Westhof, M Yusupov, and G Yusupova. A new understanding of the decoding principle on the ribosome. *Nature*, 484:256–259, 2012.

4. A Korostelev, Sergei Trakhanov, M Laurberg, and H F Noller. Crystal structure of a 70S ribosome-tRNA complex reveals functional interactions and rearrangements. *Cell*, 126(6):1065–77, Sep 2006.

5. JA Dunkle, L Wang, M B Feldman, A Pulk, V B Chen, G J Kapral, J Noeske, J S Richardson, S C Blanchard, and J H D Cate. Structures of the bacterial ribosome in classical and hybrid states of tRNA binding. *Science*, 332(6032):981–4, May 2011.

6. J Frank, H Gao, J Sengupta, N Gao, and D J Taylor. The process of mRNA-tRNA translocation. *Proc Nat Acad Sci USA*, 104(50):19671–8, Dec 2007.

7. I Wohlgemuth, C Pohl, J Mittelstaet, A L Konevega, and M V Rodnina. Evolutionary optimization of speed and accuracy of decoding on the ribosome. *Phil Trans Royal Soc B: Biol Sci*, 366(1580):2979–86, Oct 2011.

8. M Johansson, M Lovmar, and M Ehrenberg. Rate and accuracy of bacterial protein synthesis revisited. *Curr Op Microbio*, 11(2):141–147, 2008.

9. RA Marshall, CE Aitken, M Dorywalska, and JD Puglisi. Translation at the single-molecule level. *Annu Rev Biochem*, 77:177–203, Jan 2008.

10. S C Blanchard. Single-molecule observations of ribosome function. *Curr Op Struct Biol*, 19:103–109, Feb 2009.

11. M M Yusupov, G Z Yusupova, A Baucom, K Lieberman, T N Earnest, J H Cate, and H F Noller. Crystal structure of the ribosome at 5.5 a resolution. *Science*, 292(5518):883–96, May 2001.

12. F Tama, Mikel Valle, J Frank, and C L Brooks. Dynamic reorganization of the functionally active ribosome explored by normal mode analysis and cryo-electron microscopy. *Proc Nat Acad Sci USA*, 100(16):9319–23, Aug 2003.

13. Maria Selmer, Christine M Dunham, Frank V Murphy, Albert Weixlbaumer, Sabine Petry, Ann C Kelley, J R Weir, and V Ramakrishnan. Structure of the 70s ribosome complexed with mRNA and tRNA. *Science*, 313(5795):1935–42, Sep 2006.

14. J Frank and CMT Spahn. The ribosome and the mechanism of protein synthesis. *Rep Prog Phys*, 69(5):1383–1417, Jan 2006.

15. Wei Zhang, M Kimmel, C M T Spahn, and Pawel A Penczek. Heterogeneity of large macromolecular complexes revealed by 3D cryo-EM variance analysis. *Structure*, 16(12):1770–6, Dec 2008.

16. A Korostelev, D N Ermolenko, and H F Noller. Structural dynamics of the ribosome. *Curr Opin Chem Biol*, 12(6):674–83, Dec 2008.

17. Xabier Agirrezabala, Jianlin Lei, Julie L Brunelle, Rodrigo F Ortiz-Meoz, Rachel Green, and J Frank. Visualization of the hybrid state of tRNA binding promoted by spontaneous ratcheting of the ribosome. *Molecular Cell*, 32(2):190–7, Oct 2008.

18. W Zhang, J A Dunkle, and J H D Cate. Structures of the ribosome in intermediate states of ratcheting. *Science*, 325(5943):1014–7, Aug 2009.

19. Yong-Gui Gao, Maria Selmer, Christine M Dunham, Albert Weixlbaumer, Ann C Kelley, and V Ramakrishnan. The structure of the ribosome with elongation factor G trapped in the posttranslocational state. *Science*, 326(5953):694–9, Oct 2009.

20. Atsushi Matsumoto and Hisashi Ishida. Global conformational changes of ribosome observed by normal mode fitting for 3D cryo-EM structures. *Structure (London, England : 1993)*, 17(12):1605–13, Dec 2009.

21. AH Ratje, J Loerke, A Mikolajka, M Brünner, P W Hildebrand, AL Starosta, A Dönhöfer, SR Connell, PFucini, T Mielke, P C Whitford, J N Onuchic, Y Yu, K Y Sanbonmatsu, RK Hartmann, PA Penczek, D N Wilson, and C M T Spahn. Head swivel on the ribosome facilitates translocation by means of intra-subunit tRNA hybrid sites. *Nature*, 468(7324):713–716, Dec 2010.

22. N Fischer, A L Konevega, W Wintermeyer, M V Rodnina, and H Stark. Ribosome dynamics and tRNA movement by time-resolved electron cryomicroscopy. *Nature*, 466(7304):329–33, Jul 2010.

23. Jie Fu, James B Munro, S C Blanchard, and J Frank. Cryoelectron microscopy structures of the ribosome complex in intermediate states during trna translocation. *Proc Nat Acad Sci Usa*, 108(12):4817–21, Mar 2011.

24. Robyn Hickerson, Zigurts K Majumdar, Albion Baucom, R M Clegg, and H F Noller. Measurement of internal movements within the 30S ribosomal subunit using Förster resonance energy transfer. *J Mol Biol*, 354(2):459–72, Nov 2005.

25. D N Ermolenko, P Clint Spiegel, Zigurts K Majumdar, Robyn P Hickerson, R M Clegg, and H F Noller. The antibiotic viomycin traps the ribosome in an intermediate state of translocation. *Nature Structural & Molecular Biology*, 14(6):493–7, Jun 2007.

26. James B Munro, Roger B Altman, Chang-Shung Tung, Jamie H D Cate, K Y Sanbonmatsu, and S C Blanchard. Spontaneous formation of the unlocked state of the ribosome is a multistep process. *Proc Nat Acad Sci USA*, 107(2):709–14, Jan 2010.

27. D Moazed and H F Noller. Intermediate states in the movement of transfer RNA in the ribosome. *Nature*, 342(6246):142–8, Nov 1989.

28. S C Blanchard, H D Kim, R L Gonzalez, J D Puglisi, and S Chu. tRNA dynamics on the ribosome during translation. *Proc Nat Acad Sci USA*, 101(35):12893–8, Aug 2004.

29. James B Munro, Roger B Altman, Nathan O'Connor, and S C Blanchard. Identification of two distinct hybrid state intermediates on the ribosome. *Molecular Cell*, 25(4):505–517, Jan 2007.

30. P Clint Spiegel, D N Ermolenko, and H F Noller. Elongation factor G stabilizes the hybrid-state conformation of the 70S ribosome. *RNA*, 13(9):1473–82, Sep 2007.

31. D N Ermolenko, Zigurts K Majumdar, Robyn P Hickerson, P Clint Spiegel, R M Clegg, and H F Noller. Observation of intersubunit movement of the ribosome in solution using fret. *J Mol Biol*, 370(3):530–40, Jul 2007.

32. P V Cornish, D N Ermolenko, H F Noller, and T Ha. Spontaneous intersubunit rotation in single ribosomes. *Mol Cell*, 30(5):578–88, Jun 2008.

33. JB Munro, KY Sanbonmatsu, CMT Spahn, and SC Blanchard. Navigating the ribosome's metastable energy landscape. *Trends Biochem Sci*, 34:390–400, Jul 2009.

34. LY Wang, A Pulk, MR Wasserman, MB Feldman, RB Altman, JHD Cate, and SC Blanchard. Allosteric control of the ribosome by small-molecule antibiotics. *Nat Struct Mol Biol*, 19:957–963, 2012.

35. P C Whitford, A Ahmed, Y Yu, S P Hennelly, F Tama, C M T Spahn, J N Onuchic, and K Y Sanbonmatsu. Excited states of ribosome translocation revealed through integrative molecular modeling. *Proc Nat Acad Sci USA*, 108:18943–18948, Nov 2011.

36. Z Guo, and HF Noller. Rotation of the head of the 30S ribosomal subunit during mRNA translocation. *Proc Nat Acad Sci USA*, 109:20391–20394, 2012.

37. K Bergemann, and KH Nierhaus. Spontaneous, Elongation Factor-G independent translocation of Escherichia-coli ribosomes *J Biol Chem*, 258:5105–5113, 2012.

38. W Humphrey, A Dalke, and K Schulten. VMD: Visual molecular dynamics. *J Mol Graph*, 14(1):33–38, FEB 1996.

39. PC Whitford, JN Onuchic, and KY Sanbonmatsu. Connecting energy landscapes with experimental rates for aminoacyl-tRNA accommodation in the ribosome. *J Am Chem Soc*, 132:13170–13171, Aug 2010.

40. A E García, J A Krumhansl, and H Frauenfelder. Variations on a theme by Debye and Waller: from simple crystals to proteins. *Proteins*, 29(2):153–60, Oct 1997.
